# User Experience of Intraoral Scanners in Dentistry: Transnational Questionnaire Study

**DOI:** 10.1016/j.identj.2023.04.002

**Published:** 2023-05-05

**Authors:** Ahmad Al-Hassiny, Dániel Végh, Dorottya Bányai, Ádám Végh, Zoltán Géczi, Judit Borbély, Péter Hermann, Tamás Hegedüs

**Affiliations:** a Institute of Digital Dentistry, Wellington, New Zealand; b Department of Prosthodontics, Semmelweis University, Budapest, Hungary; c Department of Pediatric Dentistry and Orthodontics, Semmelweis University, Budapest, Hungary; d Department of Oral Diagnostics, Semmelweis University, Budapest, Hungary

**Keywords:** Digital dentistry, Intraoral scanner, Survey, User experience

## Abstract

**Introduction:**

Intraoral scanners (IOS) are continuing to gain popularity in clinical dentistry, replacing the traditional impression-taking and related technology. Despite their increasing importance, there are few data on the utility and usage of IOS amongst dentists. This study investigates the user experience of IOS technology as well as the perceived quality of a variety of IOS used by dental clinicians worldwide.

**Methods:**

An online survey of 1072 dentists was conducted to elicit data on the number of individual IOS used, their accessibility, the maintenance fees, and the programmes used. The first part of the questionnaire included demographic data and related questions, whilst the second part focussed on the specific IOS used by the respondents and the satisfaction with their scanners.

**Results:**

We surveyed 1072 respondents from 109 different countries. More than three-quarters of the survey cohort (78.8%) use IOS in their daily work, whilst 21.17% do not. The average number of scanners owned by the respondents was 1.5 (±0.9), and in total, the cohort used 36 different types of IOS. More than one-third (38.6%) of the respondents used computer-aided design (CAD) software as well. As for the frequency of IOS usage, 51.5% used the system on a daily basis, 28.2% did so 2 to 3 times a week, and 10.0% did so once a week. Overall, the top 3 IOS used by the cohort were Medit i700 followed by wireless Medit i700 and Dentsply Sirona Primescan.

**Conclusions:**

This study describes, for the first time, the IOS user experience in an international cohort. More than 75% of the respondents used IOS on a daily basis in their practice, whilst Medit and Dentsply Sirona brands were the most popular scanners amongst the group. It appears that digital impression-taking technology is universal, and digital workflow in dentistry will continue to grow.

## Introduction

Computer-aided design/computer-aided manufacturing (CAD/CAM) technology was introduced to dentistry in the 1970s.^1^ Prior to that, dental professionals mainly used traditional manual techniques for various dental procedures such as impression-taking and fabrication of prostheses. These procedures are labourious, cumbersome, and relatively inaccurate compared with the newer technologies.^2^

Intraoral scanning (IOS) is a newer technology widely used with CAD/CAM for milling machines (subtractive technology) and 3D printers (additive technology). Using these tools, the fabrication of a prosthesis can be digitally performed in a virtual environment. Currently, both the IOS and CAD/CAM technology are widespread and highly popular in virtually every branch of dentistry including restorative dentistry, endodontics, prosthodontics, orthodontics, implantology, and oral surgery.^3^, ^4^, ^5^, ^6^, ^7^, ^8^

The procedure of IOS begins by taking a direct optical/digital measurement of the patient's teeth or gums. This creates a 3D picture of the surface topography of the target teeth and the gingival contours, the antagonistic tooth/teeth, and/or the state of the dental occlusion.^9^ The IOS method has both advantages and disadvantages. The major disadvantages most commonly mentioned are its purchase and the management costs and the operator error of inexperienced individuals.^10^ On the other hand, the main advantages of IOS are reduction in patient discomfort, time efficiency, simplicity of operative steps, and the feasibility of better communication amongst the dentist, dental technician, and patients, particularly in distant locations.^11^, ^12^, ^13^

Although there are numerous articles in the literature on the accuracy of IOS technology, few data are available on the user experience of the technical procedure. Hence, we conducted an online questionnaire survey on IOS user experience and the qualitative aspects of the technology in a large international cohort of dental practitioners.

## Materials and methods

The research method was based on a previous study of ours, which aimed to discover the user experience of 3D printing in dentistry.^14^ Due to the limitations of the previous study, further modifications were incorporated to provide better outcomes in the current study. It has been clearly shown that focus groups enhance the validity of questionnaires by highlighting user concerns and providing feedback that would otherwise have been missed; thus, our aim was to establish a focus group–related questionnaire.^15^^,^^16^

A total of 1751 dentists, dental assistants, dental hygienists, dental specialists, dental students, dental technicians, lab workers, industry members, and office staff members from 109 countries were asked to complete an online questionnaire. A free web survey form of the Institute of Digital Dentistry (Wellington, New Zealand), which was accessible by computer, tablet, or cell phone, was distributed amongst the respondents and the responses were received during a 1-month period between October and November 2022. The research team shared the survey through the newsletter of the Institute of Digital Dentistry (9 Hillary Court, Naenae, Lower Hutt 5010, New Zealand) and on Instagram, Facebook, and LinkedIn social media platforms.^17^ The study was conducted by the Declaration of Helsinki Ethical Principles and Good Clinical Practices. Participation was voluntary. Ethical approval was not applicable.

The 2-part questionnaire was in English, which might have limited the participation of other language speakers. Part 1 of the questionnaire included 18 questions on the demographic features of respondents and their experience with IOS. Part 2 comprised 16 specific questions related to the IOS technology that the participant chose to review. The questionnaire is shown in Table 1.

**Table 1:** The questionnaire used in the study.

Part 1: Demographic and other data	
1.	What is your name?
2.	In what country do you live?
3.	What is your email address?
4.	What describes your profession best?
5.	Have you ever used an intraoral scanner for dental work?
6.	How long have you used intraoral scanners?
7.	How many IOS do you have in your office/clinic?
8.	How would you describe your experience level with IOS and digital dentistry?
9.	Have you ever tried using your intraoral scanner to make a digital impression for fixed prosthetic appliance?
10.	Do you think digital technology is more accurate than traditional casting?
11.	Do you think digital technology may be more accurate in the future?
12.	Do you carry out any CAD (design) in-house?
13.	Were you involved in the purchase of the design software?
14.	How much did this design software cost to buy in USD?
15.	Can you upgrade the software for free of charge?
16.	Do you carry out any CAM (manufacturing) in-house?
17.	Which milling machine(s) do you use?
18.	Which 3D printer(s) do you use?
Part 2: Feedback on IOS technology 4	
1.	Add your intraoral scanner
2.	Were you involved in the purchase of the scanner?
3.	Did you participate in any education or course before you bought the scanner?
4.	What was the main reason you chose this intraoral scanner?
5.	How easy was the process to buy this scanner?
6.	In what price range did you purchase your intraoral scanner in USD?
7.	How satisfied are you with the price of the scanner?
8.	How long did it take to ship the intraoral scanner after the purchase?
9.	Did the manufacturer or dealer provide education or course (in person) with the purchase of your scanner?
10.	How satisfied are you with the training course of the scanner?
11.	How often do you use this intraoral scanner?
12.	How satisfied are you overall with this intraoral scanner?
13.	How satisfied are you with the speed of the scanner?
14.	How satisfied are you with the support service of the scanner?
15.	What is the annual cost of the intraoral scanner service and support in USD? ($/year)
16.	What is the main disadvantage of this system/intraoral scanner?

Exclusion criteria included incomplete questionnaire or responses from nondental professionals, incomplete questionnaires, and multiple or duplicate responses from the same email address.

The online data were collected and stored using Microsoft Excel and the data analysis was performed using Prism version 8.4.2. (GraphPad Software Inc.) software. The data were reported as means, standard deviations (SDs), ranges, or absolute numbers with percentages.

Data collation was done by a member of the research team (DV). The storage and collation of the data was done using Microsoft Excel. The datasets used and/or analysed during the current study are available from the corresponding author upon reasonable request.

## Results

In total, our study received 1751 responses. However, 38.8% (n=679) of these were excluded, as they were administrative staff (5.5%, n=96), industry experts (4.6%; n=80), and office staff (0.9%; 16) not belonging to the dental community or were partially completed questionnaires (33.3%; n=583). Finally, a total of 61.2% (n=1072) responses were analysed from the respondents from 109 different countries.

**Fig. 1:**
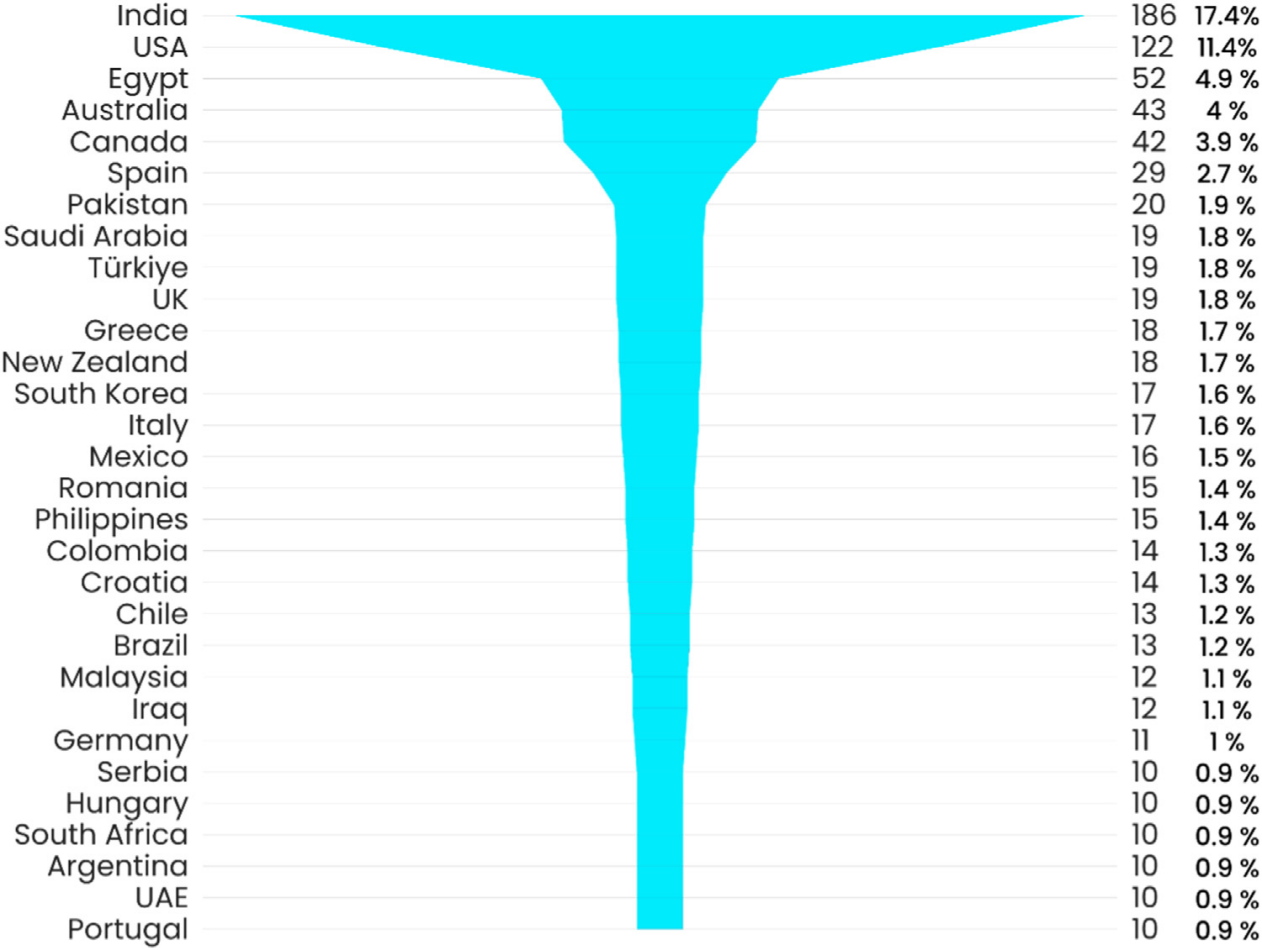
Country-wide distribution of respondents (countries with fewer than 10 responders are excluded).

The top 5 responses were from India (17.4%; n=186), the United States (11.4%; n=122), Egypt (4.9%; n=52), Australia (4%; n=43), and Canada (3.9%; n=42; Figure 1). These respondents comprised 75.8% (n=813) general dentists, 16.2% (n=174) dental specialists, 4.7% (n=50) dental technicians, 2.3% (n=25) dental students, 0.7% (n=7) dental assistants, and 0.3% (n=3) dental hygienists (Figure 1).

As for the responses, 81.9% (n=878) of the respondents considered that, with the current technology, IOS are more accurate than traditional casting methods, whilst 18.1% (n=194) believed that traditional casting is more accurate. Only 0.9% (n=10) of the respondents think digital technology will not be more accurate in the future. More than three-quarters of the respondents (78.8%; n=845) have used IOS in their daily work, and 21.2% (n=227) have not.

Regarding the period of IOS experience, out of the 78.8% (n=845) of the respondents who use IOS daily, 17.9% (n=151) used IOS for more than 5 years, 12.9% (n=109) for 3 to 5 years, 34.3% (n=290) for 1 to 3 years, 25.3% (n=214) for less than 1 year, and 9.6% (n=81) for less than 1 month.

The mean number of scanners owned by the respondents were 1.5 (±0.9). Whilst 24.7% (n=209) of the respondents considered themselves to be experts and 29.6% (n=250) beginners in the use of IOS technology, 45.7% (n=386) thought they were intermediate-level users. More than three-quarters (85.6%; n=723) used digital impressions with IOS for fabricating fixed prosthetic appliances.

**Fig. 2:**
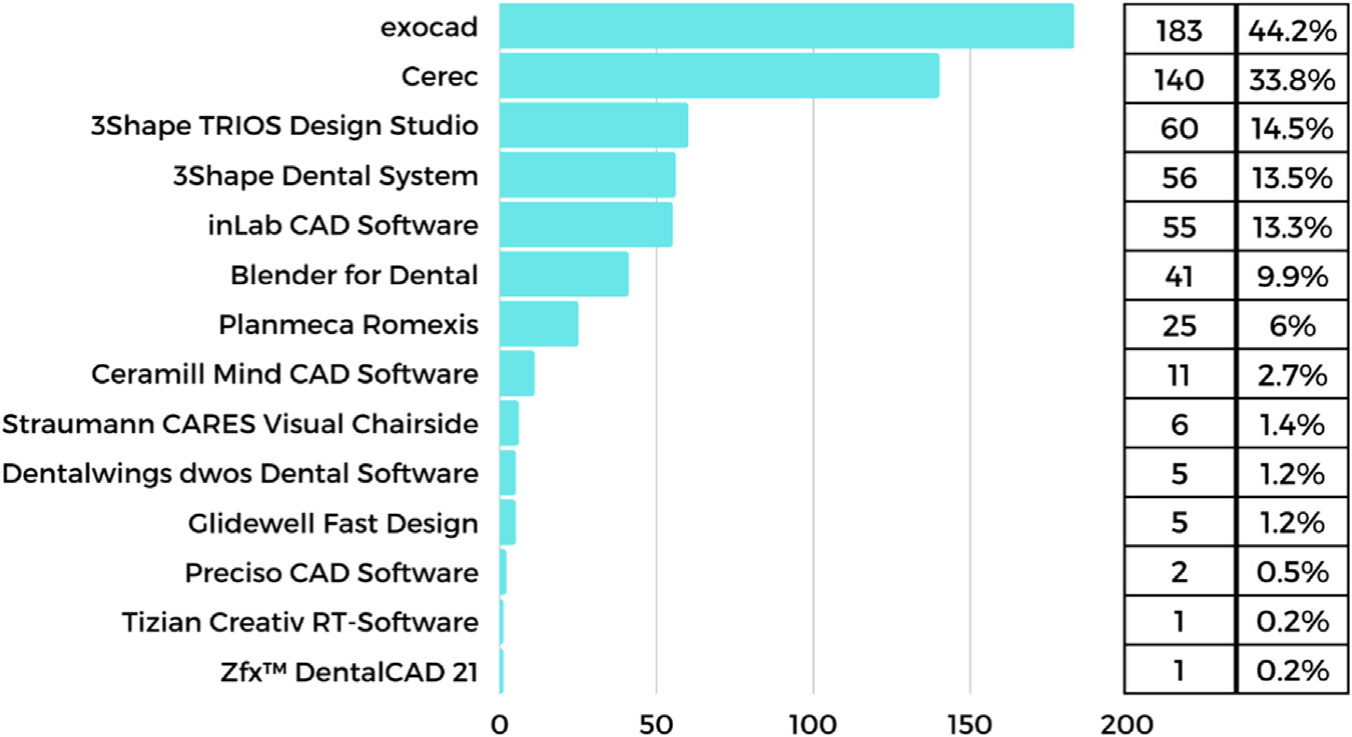
The type of computer-aided design (CAD) software used by the respondents and their popularity.

More than one-third of the respondents (38.62%; n=414) carry out CAD using in-house software. The respondents had the opportunity to indicate various multiple design software programmes they use, and the most common are shown in Figure 2.

**Fig. 3:**
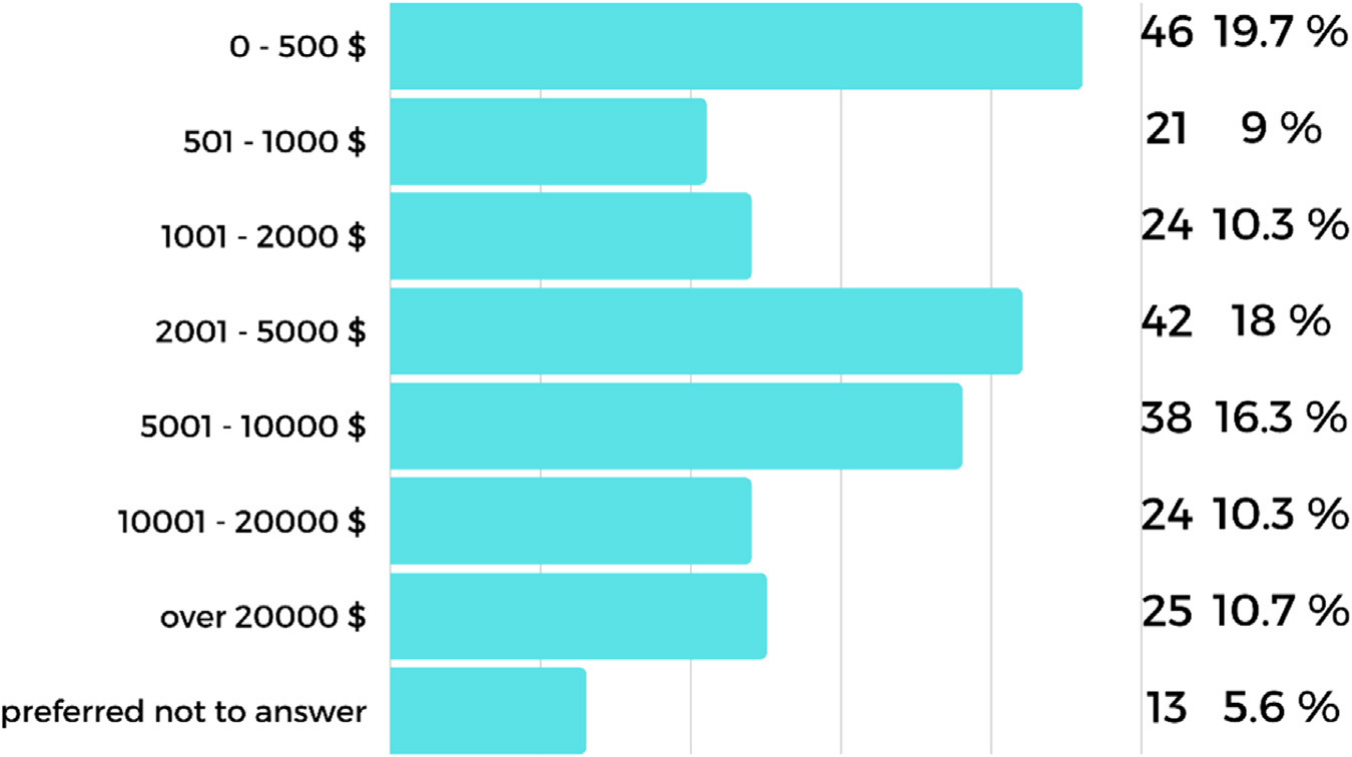
The cost of computer-aided design (CAD) software used by the respondents.

A total of 56.3% (n=233) of those who used CAD software purchased these for in-house use. Accordingly, the software costs ranged from US$0 USD (free software provided by some distributors) to more than US$20,000 (Figure 3).

Two-fifths (40.3%; n=94) of the CAD users were allowed to upgrade the software for free. Interestingly, 31.5% of the respondents (n=338) had CAM tools in-house and 11.8% (n=126) of them had a milling machine and a 3D printer; 9.8% (n=105) have only a milling machine, and 10% (n=107) have only a 3D printer.

Overall, the respondents used 36 different IOS (Table 2). Regarding the frequency of the usage of IOS by the cohort, 49.5% (n=554) used the system daily, 27.1% (n=303) did so 2 to 3 times per week, 9.7 % (n=108) once a week, 4.6% (n=51) 2 to 3 times a month, 3.6% (n=40) once a month, and 5.6% (n=63) less than once a month.

**Table 2:** The quality of the different intraoral scanners (IOS) as evaluated by the responders.

Type of intraoral scanner	No.	Overall	Speed	Support	Accessibility*	Price*
Medit i700	179	4.50	4.47	4.30	4.27	4.14
3Shape TRIOS 3	162	4.19	4.22	3.70	4.02	3.26
Dentsply Sirona Primescan	121	4.49	4.52	3.88	4.23	3.51
CEREC Omnicam	106	3.68	3.46	3.50	3.89	3.10
Medit i500	106	4.24	3.86	4.02	4.37	3.95
3Shape TRIOS 4	60	4.35	4.38	3.72	4.09	3.55
iTero Element 2	42	3.76	3.62	3.95	3.86	3.24
Medit i600	39	4.41	4.31	4.31	4.22	4.22
Shining 3D Aoralscan 3	39	4.15	4.44	4.05	4.35	4.12
Medit i700 Wireless	31	4.77	4.77	4.61	4.45	4.55
iTero Element 5D Plus	28	4.25	4.29	4.04	4.32	3.36
3DISC Heron IOS	25	3.64	3.52	3.68	4.29	4.00
Dexis Carestream CS 3600	17	3.47	3.18	3.29	4.20	3.00
iTero Element 5D	17	3.94	4.06	3.88	4.50	3.17
Straumann Virtuo Vivo	16	3.86	3.56	3.94	4.44	4.44
Planmeca Emerald S	13	4.00	3.92	4.31	4.17	3.17
Eighteeth Helios 600	12	4.25	4.25	4.00	4.38	4.13
iTero Element Flex	11	4.27	4.36	4.27	4.60	4.00
Panda P2	11	4.09	4.09	3.91	4.22	4.33
Planmeca Emerald	11	3.90	3.82	4.36	4.20	3.30
3Shape TRIOS 5	10	4.00	3.90	3.70	3.50	3.17

⁎ Only those who purchased the IOS are included in the table; fewer than 10 type systems evaluated were excluded.

Over one-half (68.6%; n=768) of the respondents were actively involved in the purchase of the IOS, and 40.1% (n=457) of them participated in education or courses before purchasing the system. The main reasons for purchasing the selected scanner were recommendations by colleagues at 26.2% (n=201), followed by the purported accuracy of the system at 19.4% (n=149), the IOS brand at 16% (n=123), the price at 15.8 (n=121), and the software design at 14.5% (n=111); 8.2% (n=63) of the respondents mentioned miscellaneous reasons.

In our questionnaire, on a scale from 1 to 5, the respondents could evaluate their satisfaction with the speed, support system, accessibility, price, and overall quality of their selected IOS (Table 2). Overall, the top 3 IOS were Medit i700 wireless (4,77), Medit i700 (4,5), and Dentsply Sirona Primescan (4,49). Regarding speed, the top 3 were Medit i700 wireless (4,77), Dentsply Sirona Primescan (4,52), and Medit i700 (4,47). The support system was the best for Medit i700 wireless (4,61) and Planmeca Emerald (4,36), and Medit i500 (4,31). We excluded devices assessed by fewer than 10 respondents in the hierarchy rankings.

Accessibility was surmised to be the best for iTero Element Flex (4,6), iTero Element 5D (4,5), and Medit i700 Wireless (4,45). In the category of price satisfaction, the top 3 were Medit i700 wireless (4,55), Straumann Virtuo Vivo (4,44), and Panda P2 (4,33).

The main disadvantages of their selected devices as perceived by the responders were, in decreasing order of importance, price (29.8%; n=333), scan speed (13.9%; n=155), quality of the software (10.3%; n=115), support service (8%; n=89), and the training course provided (7.1%; n=80), On the other hand, 17.6% (n=197) of the users answered that there is no main disadvantage and they are delighted with their purchased IOS.

The respondents also indicated their annual costs related to maintaining their IOS (Supplement 1). A majority (53.1%; n=595) of the respondents had no maintenance cost, whilst 5.3% (n=60) spent US$101-250, 5.9% (n=66) spent US$251-500, 7.51% (n=84) spent US$500-1000, 8.31% (n=93) spent US$1001-2000, 9.4 % (n=106) spent US$2001-5000, and 2.5% (n=28) paid more than US$5000 for the maintenance of their owned IOS. (A detailed breakdown is provided in the supplementary data.)

## Discussion

Over the past few decades, digital technologies including IOS technology have revolutionised the clinical approach to medicine and dentistry. Although IOS technology is relatively widely used in dentistry, there are few regional or transnational data on the utility, usage, and user experience of this technology amongst the dental profession. The current international questionnaire study provides a glimpse of the foregoing characteristics of IOS used by dentists in more than 109 countries.

Our aim was to learn more about the user experience, using social networks such as Institute of Digital Dentistry (IDD) newsletter, where colleagues from around the world are educated on the appropriate use of IOS, 3D printing, and other digital technologies and workflows. Reaching out to these colleagues and collecting data would make it possible to understand the advantages and disadvantages of IOS technology, thus rendering valuable help for others who are planning to invest in the technology. Digital investment into this ecosystem needs proper planning, as the software and hardware are relatively expensive, whilst the disparate technologies may not cross-communicate well.

This very large (1072 responses) questionnaire study was distributed in 109 countries. In total, 239 responses (22.3%) were from Europe, 219 (20.4%) from Asia, and 197 (18.4%) from North America. Africa was in the fourth place, with 119 (11.1%) answers, and the rest of the continents comprised 298 responses. This response rate is considered to be satisfactory for such large questionnaire studies.

The majority of the responses were from India (17.4%), followed by the US (11.4%), Egypt (4.9%), Australia (4%), and Canada (3.9%). Such geographic representation incorporating Asia, the US, and Europe could therefore be construed as transnational. We believe this to be the first such transnational survey of IOS reported in the literature.

When manufacturers and distributors sell their wares, they usually highlight the advantages of the specific IOS brands. However, it appears that the disadvantages usually surface only after the purchase of the equipment, and this was clearly shown in the study as a large proportion of the responders were unhappy about the quality of the software, the support services, and the training courses provided.^18^ Hence, appropriate education and training on the management of these devices are crucial, as IOS serve several functions, from simple scanning to more complicated software and CAD functions.

One interesting revelation in our study was the large variety of IOS scanner systems and software available worldwide, as we noted more than 35 different systems used by the responders. It will be interesting to study the compatibility between the disparate software and hardware. This implies that although there have been requests towards more open and compatible systems that permit communication between independent components, the manufacturers do not pay much heed to such requests or calls. Uniformity and compatibility of software and hardware make it possible to create and transfer images to CAD devices using a wide range of image acquisition devices.^19^, ^20^, ^21^

We noted that more than one-half of the respondents used their IOS system on a daily basis, and more than one-quarter used it 2 to 3 times a week, implying that scanner use is becoming universally common. Our data imply that in future this will be a part of routine dentistry due to their simplicity and effectiveness. It is tempting to speculate that together with artificial intelligence systems that are beginning to enter the medical and dental field, it is likely that many far-reaching technological advances in IOS may occur in the future.

The survey also indicated that the maintenance cost of the IOS varied considerably from a few hundred dollars to more than $1000 per year in different countries as well as in the same country. Such costs depend on a number of factors such as the frequency of use and the IOS brand, and these are important considerations to bear in mind when purchasing a new IOS and software. Further comparative studies should be undertaken to provide relevant data to the dental community in this context. Currently, the only data available are derived from the manufacturers, excluding the users in the field.

In conclusion, our questionnaire study describes, for the first time, the IOS user experience in an international cohort of more than 1000 participants. More than 75% of the respondents used IOS on a daily basis in their practice, whilst 2 specific brands (Medit and Dentsply Sirona) were the frontrunners amongst the respondents. It appears that digital impression-taking technology is universal, and digital workflow will continue to grow in dentistry.

## Appendix Supplementary materials

Image, application 1

## Footnotes

Supplementary material associated with this article can be found in the online version at doi:10.1016/j.identj.2023.04.002.
