# Supplementary material for: User Experience of Intraoral Scanners in Dentistry: Transnational Questionnaire Study
Source: Int Dent J. 2023 May 5;73(5):754–9. doi: 10.1016/j.identj.2023.04.002 (PMC10509442; doi:10.1016/j.identj.2023.04.002)
Supplement: Supplementary file 1 [file mmc1.docx]

**Supplement 1.**

The annual maintenance costs of the reported brands IOS.

|  | **0 USD** | **1-100 USD** | **101-250 USD** | **251-500 USD** | **500-1000 USD** | **1001-2000 USD** | **2001-5000 USD** | **Over 5000 USD** |
| --- | --- | --- | --- | --- | --- | --- | --- | --- |
| **3DISC Heron IOS** | **17** | **3** | **0** | **0** | **2** | **2** | **0** | **1** |
| **3Shape TRIOS 3** | **43** | **7** | **12** | **10** | **24** | **34** | **26** | **6** |
| **3Shape TRIOS 4** | **20** | **1** | **3** | **5** | **10** | **10** | **11** | **0** |
| **3Shape TRIOS 5** | **2** | **2** | **1** | **1** | **0** | **2** | **1** | **1** |
| **AlliedStar AS 100** | **4** | **0** | **0** | **0** | **0** | **0** | **0** | **0** |
| **CEREC Omnicam** | **48** | **8** | **8** | **5** | **9** | **13** | **10** | **5** |
| **Glidewell fastscan.io** | **1** | **1** | **0** | **0** | **0** | **0** | **0** | **0** |
| **iTero Element 2** | **6** | **3** | **1** | **5** | **4** | **8** | **14** | **1** |
| **iTero Element 5D** | **3** | **1** | **0** | **1** | **4** | **2** | **6** | **0** |
| **iTero Element 5D Plus** | **4** | **2** | **2** | **1** | **4** | **2** | **11** | **2** |
| **iTero Element Flex** | **5** | **1** | **0** | **2** | **1** | **0** | **1** | **1** |
| **Ivoclar VivaScan** | **5** | **1** | **2** | **0** | **0** | **0** | **0** | **0** |
| **Kulzer cara i500** | **1** | **0** | **0** | **0** | **0** | **0** | **0** | **0** |
| **Kulzer cara i700** | **1** | **0** | **0** | **0** | **0** | **0** | **0** | **0** |
| **Launca DL-206** | **7** | **0** | **0** | **1** | **0** | **0** | **0** | **0** |
| **Medit i500** | **76** | **13** | **6** | **4** | **3** | **0** | **0** | **4** |
| **Medit i600** | **29** | **4** | **2** | **3** | **0** | **0** | **1** | **0** |
| **Medit i700** | **130** | **25** | **8** | **7** | **5** | **0** | **3** | **1** |
| **Medit i700 Wireless** | **21** | **5** | **2** | **1** | **0** | **2** | **0** | **0** |
| **Panda P2** | **10** | **0** | **1** | **0** | **0** | **0** | **0** | **0** |
| **Panda P3** | **1** | **1** | **0** | **0** | **0** | **1** | **0** | **0** |
| **Planmeca Emerald** | **6** | **0** | **0** | **0** | **0** | **4** | **1** | **0** |
| **Planmeca Emerald S** | **12** | **0** | **0** | **0** | **1** | **0** | **0** | **0** |
| **RunYes 3DS** | **2** | **0** | **1** | **0** | **0** | **0** | **0** | **0** |
| **RunYes 3DS 2.0** | **2** | **1** | **0** | **0** | **0** | **0** | **0** | **0** |
| **Shining 3D Aoralscan 1** | **2** | **0** | **1** | **0** | **0** | **0** | **0** | **0** |
| **Shining 3D Aoralscan 2** | **3** | **0** | **0** | **2** | **0** | **0** | **0** | **0** |
| **Shining 3D Aoralscan 3** | **36** | **2** | **1** | **0** | **0** | **0** | **0** | **0** |
| **Straumann Virtuo Vivo** | **10** | **2** | **1** | **2** | **1** | **0** | **0** | **0** |
| **Vatech EzScan** | **5** | **0** | **1** | **0** | **0** | **0** | **1** | **0** |
